# 2-Methyl-4-phenyl-3,4-dihydro­quinazoline

**DOI:** 10.1107/S1600536811009664

**Published:** 2011-03-19

**Authors:** Arto Valkonen, Erkki Kolehmainen, Anna Zakrzewska, Agnieszka Skotnicka, Ryszard Gawinecki

**Affiliations:** a University of Jyväskylä, Department of Chemistry, PO Box 35, FIN-40014 Jyväskylä, Finland; b University of Technology and Life Sciences, Department of Chemistry, Seminaryjna 3, PL-85-326 Bydgoszcz, Poland

## Abstract

The title compound, C_15_H_14_N_2_, was formed during the lithia­tion of 2-methyl­quinazoline with phenyl­lithium followed by hydrolysis of the inter­mediate lithium 2-methyl-4-phenyl-4*H*-quinazolin-3-ide. NMR spectra as well as single-crystal X-ray structural data indicate that the reaction product to have the same structure in chloro­form solution as in the crystalline state. The phenyl substituent is twisted out of the plane of the 3,4-dihydro­quinazoline ring system by 86.47 (7)°. In the crystal, inter­molecular N—H⋯N inter­actions connect the mol­ecules into infinite chains.

## Related literature

For organolithium compounds and lithia­tion, see: Gawinecki *et al.* (2006 ▶); Kolehmainen *et al.* (2000 ▶); Wakefield (1976 ▶); Armarego (1967 ▶). For previous characterizations of the title compound, see: Suri *et al.* (1993 ▶). For related structures, see: Rajnikant *et al.* (2002 ▶).
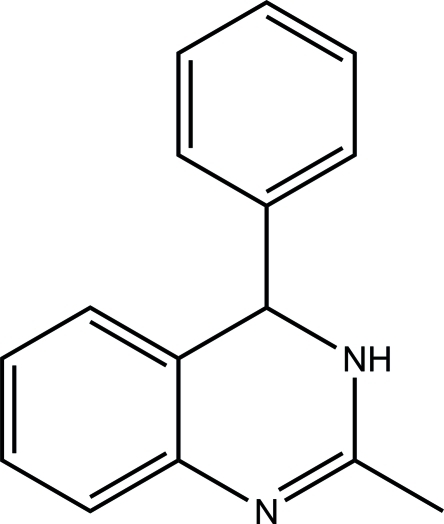


## Experimental

### 

#### Crystal data

C_15_H_14_N_2_
*M* *_r_* = 222.28
Trigonal, 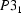

*a* = 9.5600 (4) Å
*c* = 11.2569 (5) Å
*V* = 890.97 (7) Å^3^
*Z* = 3
Mo *K*α radiation
μ = 0.07 mm^−1^
*T* = 123 K
0.35 × 0.13 × 0.12 mm

#### Data collection

Bruker–Nonius KappaCCD with APEXII detector diffractometer
6729 measured reflections
1468 independent reflections
1215 reflections with *I* > 2σ(*I*)
*R*_int_ = 0.068

#### Refinement

*R*[*F* ^2^ > 2σ(*F* ^2^)] = 0.046
*wR*(*F* ^2^) = 0.095
*S* = 1.06
1468 reflections
155 parameters
1 restraint
H-atom parameters constrained
Δρ_max_ = 0.19 e Å^−3^
Δρ_min_ = −0.19 e Å^−3^

### 

Data collection: *COLLECT* (Bruker, 2008 ▶); cell refinement: *DENZO-SMN* (Otwinowski & Minor, 1997 ▶); data reduction: *DENZO-SMN*; program(s) used to solve structure: *SIR2004* (Burla *et al.*, 2005 ▶); program(s) used to refine structure: *SHELXL97* (Sheldrick, 2008 ▶); molecular graphics: *ORTEP-3* (Farrugia, 1997 ▶) and *Mercury* (Macrae, *et al.*, 2008 ▶); software used to prepare material for publication: *SHELXL97*.

## Supplementary Material

Crystal structure: contains datablocks global, I. DOI: 10.1107/S1600536811009664/im2274sup1.cif

Structure factors: contains datablocks I. DOI: 10.1107/S1600536811009664/im2274Isup2.hkl

Additional supplementary materials:  crystallographic information; 3D view; checkCIF report

## Figures and Tables

**Table 1:** Hydrogen-bond geometry (Å, °)

*D*—H⋯*A*	*D*—H	H⋯*A*	*D*⋯*A*	*D*—H⋯*A*
N3—H3⋯N1^i^	0.88	2.04	2.908 (3)	169

Symmetry code: (i) 
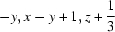
.

**Fig. 1.**
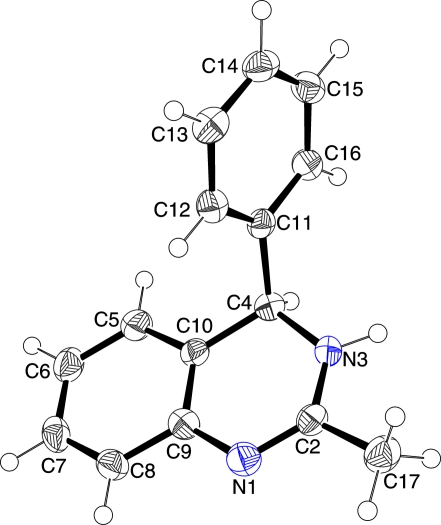
Molecular structure of the title compound showing the atom-labeling scheme. Displacement ellipsoids are drawn at the 50% probability level. H atoms are represented by circles of arbitrary size.

**Fig. 2.**
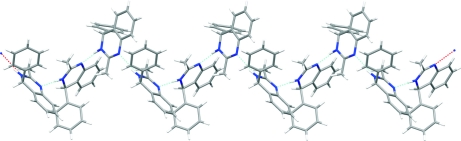
Part of the helical chain. Right-handed arbitrary presentation.

## supplementary crystallographic information

### Comment

Addition of phenyllithium to 2-methylquinazoline takes place exclusively at the 3,4-position (neither 2-methyl-2-phenyl-1,2-dihydroquinazoline nor 2-methyl-4-phenyl-1,4-dihydroquinazoline were detected in the reaction mixture). Susceptibility of quinazolines to undergo the nucleophilic addition to their 3,4-double bonds has been reported earlier (Suri *et al.*, 1993). It is also known that 4-substituted 3,4-dihydroquinazolines can be generated from quinazolines when treated with organometallic compounds (Armarego, 1967). Furthermore, low susceptibility of 2-methyl group to lithiation precludes 2-methylquinazoline to be used as a starting material in syntheses of the important C*_exo_*-substituted 2-methylquinazolines (Wakefield, 1976; Kolehmainen *et al.*, 2000; Gawinecki *et al.*, 2006).

In crystalline state the title compound shows the 3,4-dihydroquinazoline moiety to be planar (Fig. 1). The phenyl substituent is twisted out of plane of the moiety by 86.47 (7) °, which is rather close to the twist (79.3 (1) °) found in 2-methyl-4-phenyl-3,4-dihydroquinazolinium chloride (Rajnikant *et al.*, 2002).  Intermolecular N3—H···N1 hydrogen bonds (-*y*, *x*-*y* + 1, *z* + 1/3 direction) define the supramolecular structure and connect the molecules to infinite helical chains (Fig. 2). Unfortunately, no reliable determination of the absolute structure (or handedness of helix) is possible by X-ray crystallography.

### Experimental

A solution of 2-methyl-quinazoline (10.09 g, 0.07 mol) in absolute ethyl ether (100 ml) was added dropwise with stirring to a solution of phenyllithium [obtained by a standard method starting from freshly distilled bromobenzene (15.70 g, 0.1 mol), absolute ethyl ether (0.5 *L*) and lithium (2.80 g, 0.4 mol)]. The reaction mixture was stirred at room temperature for additional 2 h and the reaction was quenched by addition of water (0.5 *L*). The organic layer was combined with the ether extracts of the water layer, dried (K_2_CO_3_) and evaporated to dryness. The crude solid product was recrystallized from ethanol to give white crystals (51%) melting at 168–170 °C [lit. mp 168–170 °C (Suri *et al.*, 1993)]. ^1^H NMR (CDCl_3_): δ (p.p.m.) = 7.26–7.34 (m, 5H, H12—H16), 7.13 (dd, 1H, H7), 7.02 (d, 1H, H8), 6.90 (dd, 1H, H6), 6.71 (d, 1H, H5), 5.67 (s, 1H, H4), 2.02 (s, 3H, CH3). ^13^C NMR (CDCl_3_): δ (p.p.m.) = 153.9 (C2), 145.3 (C11), 140.6 (C9), 128.1 (C7), 127.8 (C14), 128.7 (C13, C15), 127.3 (C12, C16), 126.7 (C5), 124.1 (C6), 123.3 (C10), 58.1 (C4), 22.5 (C17).

Suitable single crystals for X-ray diffraction were obtained by very slow evaporation of analytical sample from NMR-tube, where CDCl_3_ was used as a solvent.

### Refinement

In the absence of significant anomalous disperson effects, Friedel pairs were averaged. All H atoms were visible in electron density maps, but were calculated at their idealized positions and allowed to ride on their parent atoms at C—H distances of 0.95 Å (aromatic), 0.98 Å (methyl), 1.00 Å (methine), and N—H distance of 0.88 Å, with *U*_iso_(H) of 1.2 times *U*_eq_(C,*N*) or 1.5 times *U*_eq_(C) (methyl).

### Crystal data

**Table:**

C_15_H_14_N_2_	*D*_x_ = 1.243 Mg m^−^^3^
*M**_r_* = 222.28	Mo *K*α radiation, λ = 0.71073 Å
Trigonal, *P*3_1_	Cell parameters from 3635 reflections
Hall symbol: P 31	θ = 0.4–28.3°
*a* = 9.5600 (4) Å	µ = 0.07 mm^−^^1^
*c* = 11.2569 (5) Å	*T* = 123 K
*V* = 890.97 (7) Å^3^	Long plate, colourless
*Z* = 3	0.35 × 0.13 × 0.12 mm
*F*(000) = 354	

### Data collection

**Table:**

Bruker–Nonius KappaCCD with APEXII detector diffractometer	1215 reflections with *I* > 2σ(*I*)
Radiation source: fine-focus sealed tube	*R*_int_ = 0.068
graphite	θ_max_ = 28.2°, θ_min_ = 2.5°
Detector resolution: 9 pixels mm^-1^	*h* = −12→12
φ and ω scans	*k* = −12→12
6729 measured reflections	*l* = −12→14
1468 independent reflections	

### Refinement

**Table:**

Refinement on *F*^2^	Primary atom site location: structure-invariant direct methods
Least-squares matrix: full	Secondary atom site location: difference Fourier map
*R*[*F*^2^ > 2σ(*F*^2^)] = 0.046	Hydrogen site location: inferred from neighbouring sites
*wR*(*F*^2^) = 0.095	H-atom parameters constrained
*S* = 1.06	*w* = 1/[σ^2^(*F*_o_^2^) + (0.0321*P*)^2^ + 0.2356*P*] where *P* = (*F*_o_^2^ + 2*F*_c_^2^)/3
1468 reflections	(Δ/σ)_max_ < 0.001
155 parameters	Δρ_max_ = 0.19 e Å^−^^3^
1 restraint	Δρ_min_ = −0.19 e Å^−^^3^

### Special details

**Table:**

Geometry. All s.u.'s (except the s.u. in the dihedral angle between two l.s. planes) are estimated using the full covariance matrix. The cell s.u.'s are taken into account individually in the estimation of s.u.'s in distances, angles and torsion angles; correlations between s.u.'s in cell parameters are only used when they are defined by crystal symmetry. An approximate (isotropic) treatment of cell s.u.'s is used for estimating s.u.'s involving l.s. planes.
Refinement. Refinement of *F*^2^ against ALL reflections. The weighted *R*-factor *wR* and goodness of fit *S* are based on *F*^2^, conventional *R*-factors *R* are based on *F*, with *F* set to zero for negative *F*^2^. The threshold expression of *F*^2^ > 2σ(*F*^2^) is used only for calculating *R*-factors(gt) *etc*. and is not relevant to the choice of reflections for refinement. *R*-factors based on *F*^2^ are statistically about twice as large as those based on *F*, and *R*- factors based on ALL data will be even larger.

### Fractional atomic coordinates and isotropic or equivalent isotropic displacement parameters (Å^2^)

**Table:**

	*x*	*y*	*z*	*U*_iso_*/*U*_eq_	
N1	−0.1844 (3)	0.2606 (3)	0.0291 (2)	0.0332 (5)	
N3	−0.1137 (3)	0.4459 (3)	0.1862 (2)	0.0308 (5)	
H3	−0.1520	0.4742	0.2470	0.037*	
C2	−0.2197 (3)	0.3278 (3)	0.1150 (2)	0.0277 (6)	
C4	0.0619 (3)	0.5311 (3)	0.1691 (2)	0.0266 (6)	
H4	0.1125	0.5233	0.2447	0.032*	
C5	0.2646 (3)	0.4997 (4)	0.0469 (3)	0.0339 (7)	
H5	0.3474	0.5863	0.0910	0.041*	
C6	0.3061 (4)	0.4266 (4)	−0.0421 (3)	0.0379 (7)	
H6	0.4165	0.4634	−0.0591	0.045*	
C7	0.1854 (4)	0.2994 (4)	−0.1060 (3)	0.0362 (7)	
H7	0.2129	0.2484	−0.1667	0.043*	
C8	0.0256 (4)	0.2473 (3)	−0.0813 (2)	0.0327 (6)	
H8	−0.0563	0.1604	−0.1258	0.039*	
C9	−0.0184 (3)	0.3198 (3)	0.0081 (2)	0.0281 (6)	
C10	0.1033 (3)	0.4480 (3)	0.0727 (2)	0.0264 (6)	
C11	0.1197 (3)	0.7087 (3)	0.1459 (2)	0.0257 (6)	
C12	0.1080 (3)	0.7642 (3)	0.0349 (2)	0.0295 (6)	
H12	0.0722	0.6927	−0.0311	0.035*	
C13	0.1485 (3)	0.9247 (3)	0.0197 (3)	0.0341 (7)	
H13	0.1425	0.9627	−0.0570	0.041*	
C14	0.1977 (3)	1.0291 (4)	0.1157 (3)	0.0351 (7)	
H14	0.2219	1.1376	0.1055	0.042*	
C15	0.2112 (3)	0.9745 (3)	0.2266 (3)	0.0337 (6)	
H15	0.2470	1.0461	0.2925	0.040*	
C16	0.1724 (3)	0.8150 (3)	0.2415 (2)	0.0287 (6)	
H16	0.1820	0.7781	0.3178	0.034*	
C17	−0.3942 (3)	0.2713 (4)	0.1398 (3)	0.0384 (7)	
H17A	−0.4623	0.1767	0.0907	0.058*	
H17B	−0.4170	0.3581	0.1211	0.058*	
H17C	−0.4172	0.2424	0.2239	0.058*	

### Atomic displacement parameters (Å^2^)

**Table:**

	*U*^11^	*U*^22^	*U*^33^	*U*^12^	*U*^13^	*U*^23^
N1	0.0291 (12)	0.0346 (13)	0.0338 (13)	0.0144 (11)	−0.0030 (10)	−0.0035 (11)
N3	0.0272 (12)	0.0290 (12)	0.0297 (12)	0.0090 (10)	0.0067 (10)	−0.0042 (10)
C2	0.0270 (14)	0.0274 (14)	0.0282 (14)	0.0131 (12)	0.0016 (11)	0.0043 (11)
C4	0.0253 (13)	0.0249 (13)	0.0282 (14)	0.0114 (11)	−0.0007 (11)	0.0003 (11)
C5	0.0275 (14)	0.0318 (15)	0.0395 (17)	0.0126 (12)	0.0032 (12)	−0.0008 (12)
C6	0.0352 (16)	0.0366 (16)	0.0451 (18)	0.0204 (14)	0.0136 (14)	0.0069 (14)
C7	0.0548 (19)	0.0348 (16)	0.0307 (15)	0.0313 (15)	0.0137 (14)	0.0080 (13)
C8	0.0410 (16)	0.0273 (14)	0.0308 (15)	0.0179 (13)	0.0017 (13)	−0.0010 (12)
C9	0.0329 (15)	0.0278 (13)	0.0264 (13)	0.0174 (12)	−0.0015 (12)	0.0014 (11)
C10	0.0276 (13)	0.0230 (13)	0.0279 (14)	0.0122 (11)	0.0036 (11)	0.0032 (11)
C11	0.0196 (12)	0.0264 (13)	0.0309 (15)	0.0113 (11)	0.0030 (10)	0.0009 (11)
C12	0.0266 (14)	0.0333 (15)	0.0297 (14)	0.0158 (12)	−0.0011 (12)	−0.0010 (12)
C13	0.0306 (15)	0.0352 (16)	0.0378 (16)	0.0175 (13)	0.0008 (12)	0.0069 (13)
C14	0.0277 (15)	0.0271 (14)	0.0511 (18)	0.0141 (12)	0.0046 (13)	0.0047 (13)
C15	0.0278 (15)	0.0312 (15)	0.0416 (16)	0.0142 (12)	−0.0011 (12)	−0.0073 (13)
C16	0.0253 (13)	0.0306 (14)	0.0298 (15)	0.0137 (12)	−0.0021 (11)	−0.0030 (11)
C17	0.0282 (16)	0.0404 (17)	0.0404 (17)	0.0125 (13)	−0.0001 (13)	−0.0014 (13)

### Geometric parameters (Å, °)

**Table:**

N1—C2	1.296 (4)	C8—H8	0.9500
N1—C9	1.413 (3)	C9—C10	1.401 (4)
N3—C2	1.341 (4)	C11—C12	1.384 (4)
N3—C4	1.467 (3)	C11—C16	1.390 (4)
N3—H3	0.8800	C12—C13	1.392 (4)
C2—C17	1.500 (4)	C12—H12	0.9500
C4—C10	1.509 (4)	C13—C14	1.384 (4)
C4—C11	1.523 (3)	C13—H13	0.9500
C4—H4	1.0000	C14—C15	1.384 (4)
C5—C6	1.388 (4)	C14—H14	0.9500
C5—C10	1.394 (4)	C15—C16	1.387 (4)
C5—H5	0.9500	C15—H15	0.9500
C6—C7	1.387 (5)	C16—H16	0.9500
C6—H6	0.9500	C17—H17A	0.9800
C7—C8	1.378 (4)	C17—H17B	0.9800
C7—H7	0.9500	C17—H17C	0.9800
C8—C9	1.400 (4)		
			
C2—N1—C9	116.5 (2)	C5—C10—C9	119.3 (2)
C2—N3—C4	124.2 (2)	C5—C10—C4	119.9 (2)
C2—N3—H3	117.9	C9—C10—C4	120.8 (2)
C4—N3—H3	117.9	C12—C11—C16	119.2 (2)
N1—C2—N3	126.1 (2)	C12—C11—C4	121.9 (2)
N1—C2—C17	118.6 (2)	C16—C11—C4	118.6 (2)
N3—C2—C17	115.3 (2)	C11—C12—C13	120.2 (3)
N3—C4—C10	109.3 (2)	C11—C12—H12	119.9
N3—C4—C11	108.5 (2)	C13—C12—H12	119.9
C10—C4—C11	114.8 (2)	C14—C13—C12	120.4 (3)
N3—C4—H4	108.0	C14—C13—H13	119.8
C10—C4—H4	108.0	C12—C13—H13	119.8
C11—C4—H4	108.0	C15—C14—C13	119.6 (3)
C6—C5—C10	121.1 (3)	C15—C14—H14	120.2
C6—C5—H5	119.5	C13—C14—H14	120.2
C10—C5—H5	119.5	C14—C15—C16	120.0 (3)
C7—C6—C5	119.6 (3)	C14—C15—H15	120.0
C7—C6—H6	120.2	C16—C15—H15	120.0
C5—C6—H6	120.2	C15—C16—C11	120.6 (3)
C8—C7—C6	119.9 (3)	C15—C16—H16	119.7
C8—C7—H7	120.0	C11—C16—H16	119.7
C6—C7—H7	120.0	C2—C17—H17A	109.5
C7—C8—C9	121.3 (3)	C2—C17—H17B	109.5
C7—C8—H8	119.4	H17A—C17—H17B	109.5
C9—C8—H8	119.4	C2—C17—H17C	109.5
C8—C9—C10	118.9 (2)	H17A—C17—H17C	109.5
C8—C9—N1	118.5 (3)	H17B—C17—H17C	109.5
C10—C9—N1	122.6 (2)		
			
C9—N1—C2—N3	−1.0 (4)	N1—C9—C10—C4	−0.4 (4)
C9—N1—C2—C17	179.0 (3)	N3—C4—C10—C5	−175.0 (2)
C4—N3—C2—N1	7.2 (4)	C11—C4—C10—C5	62.8 (3)
C4—N3—C2—C17	−172.8 (3)	N3—C4—C10—C9	5.4 (3)
C2—N3—C4—C10	−8.7 (4)	C11—C4—C10—C9	−116.8 (3)
C2—N3—C4—C11	117.2 (3)	N3—C4—C11—C12	−81.0 (3)
C10—C5—C6—C7	−0.4 (4)	C10—C4—C11—C12	41.6 (3)
C5—C6—C7—C8	0.4 (4)	N3—C4—C11—C16	93.3 (3)
C6—C7—C8—C9	−0.3 (4)	C10—C4—C11—C16	−144.1 (2)
C7—C8—C9—C10	0.2 (4)	C16—C11—C12—C13	−0.1 (4)
C7—C8—C9—N1	−179.9 (2)	C4—C11—C12—C13	174.2 (2)
C2—N1—C9—C8	177.9 (3)	C11—C12—C13—C14	−1.3 (4)
C2—N1—C9—C10	−2.3 (4)	C12—C13—C14—C15	2.0 (4)
C6—C5—C10—C9	0.3 (4)	C13—C14—C15—C16	−1.3 (4)
C6—C5—C10—C4	−179.3 (3)	C14—C15—C16—C11	−0.1 (4)
C8—C9—C10—C5	−0.2 (4)	C12—C11—C16—C15	0.8 (4)
N1—C9—C10—C5	179.9 (2)	C4—C11—C16—C15	−173.7 (2)
C8—C9—C10—C4	179.4 (2)		

### Hydrogen-bond geometry (Å, °)

**Table:**

*D*—H···*A*	*D*—H	H···*A*	*D*···*A*	*D*—H···*A*
N3—H3···N1^i^	0.88	2.04	2.908 (3)	169

Symmetry codes: (i) −*y*, *x*−*y*+1, *z*+1/3.
